# Comparison of the healthcare-associated infections in intensive care units in Turkey before and during COVID-19

**DOI:** 10.1186/s43162-023-00215-2

**Published:** 2023-04-17

**Authors:** Sema Sarı, Ferhat Arslan, Sema Turan, Tuğçe Mengi, Handan Ankaralı, Ahmet Sarı, Mine Altınkaya Çavuş, Çilem Bayındır Dicle, Derya Tatlısuluoğlu, Hüseyin Arıcan, Yahya Tahta, Haluk Vahaboğlu

**Affiliations:** 1 Department of Intensive Care, Ömer Halisdemir University, Training and Research Hospital, Aşağı Kayabaşı Mah. Hastaneler Cad., Kumluca Mevki, 51100 Merkez Niğde, Turkey; 2 Department of Infectious Diseases and Clinical Microbiology, Istanbul Medeniyet University, Istanbul, Turkey; 3 Department of Intensive Care, University of Health Sciences, Ankara City Hospital, Ankara, Turkey; 4 Biostatistics and Medical Informatics Department, Medical Faculty, Istanbul Medeniyet University, Istanbul, Turkey; 5 Department of Anaesthesiology and Reanimation, Haydarpaşa Numune Training and Research Hospital, Istanbul, Turkey; 6 Department of Intensive Care, Kayseri City Hospital, Ankara, Turkey; 7 Department of Intensive Care, İstanbul Başakşehir Çam ve Sakura City Hospital, Istanbul, Turkey; 8 Department of Intensive Care, Medical Faculty, Erciyes University, Kayseri, Turkey; 9 Department of Anatomy, Institute of Health Sciences, Erciyes University, Kayseri, Turkey

**Keywords:** Bloodstream infections, Healthcare-associated infections, COVID-19, Intensive care unit, Ventilator-associated pneumonia

## Abstract

**Background:**

Secondary bacterial infections are an important cause of mortality in patients with coronavirus disease 2019 (COVID-19). All healthcare providers acted with utmost care with the reflex of protecting themselves during the COVID-19 period. We aimed to compare the rates of ventilator-associated pneumonia (VAP) and bloodstream infections (BSIs) in our intensive care units (ICUs) before and during the COVID-19 outbreak surges.

**Methods:**

This multicenter, retrospective, cross-sectional study was performed in six centers in Turkey. We collected the patient demographic characteristics, comorbidities, reasons for ICU admission, mortality and morbidity scores at ICU admission, and laboratory test data.

**Results:**

A total of 558 patients who required intensive care from six centers were included in the study. Four hundred twenty-two of these patients (males (62%), whose mean age was 70 [IQR, 58–79] years) were followed up in the COVID period, and 136 (males (57%), whose mean age was 73 [IQR, 61–82] years) were followed up in the pre-COVID period. BSI and VAP rates were 20.7 (19 events in 916 patient days) and 17 (74 events in 4361 patient days) with a −3.8 difference (*P* = 0.463), and 33.7 (31 events in 919 patient days) and 34.6 (93 events in 2685 patient days) with a 0.9 difference (*P* = 0.897), respectively. The mortality rates were 71 (52%) in pre-COVID and 291 (69%) in COVID periods.

**Conclusion:**

Protective measures that prioritize healthcare workers rather than patients and exceed standard measures made no difference in terms of reducing mortality.

## Introduction

Despite the worldwide mass vaccination strategy, coronavirusdisease 2019 (COVID-19) caused significant morbidity and mortality with emerging new vaccine-resistant variants. It worsened those with an underlying disease, such as diabetes, cancer, and neurological disorders [1, 2]. Non-invasive and/or invasive mechanical respiratory support may be required in patients with COVID-19 who develop progressive respiratory failure. Secondary bacterial infections in the intensive care unit (ICU) are an important cause of mortality in these patients [3, 4].

Corticosteroids and other immunomodulatory agents [e.g., interleukin (IL)-6, IL-1 antagonists], frequently used in patients with COVID-19, have the potential to increase nosocomial infections [5]. Preventive bundles have been developed to prevent intensive care-associated infections [6]. Strict contact precautions and hand hygiene are the main factors among these measures. All healthcare providers acted with utmost care with the reflex of protecting themselves during the COVID-19 period, especially for strict contact precautions and hand hygiene [7]. It is an important issue whether this care provides a reduction in the rates of hospital infections. There are conflicting results regarding the frequency of nosocomial infections in ICU observational studies conducted during the COVID-19 period.

In a meta-analysis evaluating bacterial co-infection and secondary bacterial infections, the burden of infections was not found statistically significant in hospitalized patients at the beginning of the pandemic [8]. We believe the dynamic process of the disease and intensive care conditions require a different perspective.

In this study, we aimed to compare the rates of ventilator-associated pneumonia (VAP) and bloodstream infections (BSIs) in our ICUs before and during the COVID-19 outbreak.

## Material and methods

### Study design and population

This multicenter, retrospective, cross-sectional study was performed in six centers in Turkey. In this study, we analyzed patients admitted to the ICU during the first wave of COVID-19 (April 1, 2020, to June 30, 2020) and the second wave of COVID-19 (October 1, 2020, to December 31, 2020) and compared them with patients admitted to the ICU before the COVID-19 pandemic (April 1, 2019, to June 30, 2019).

Randomly selected adult patients were included in the study who were hospitalized in the ICU for 3 to 28 days. We excluded pregnant women and patients who had an infection at the time of admission to the ICU or who were in the incubation period of probable nosocomial infections (48 h of ICU hospitalization).

### Data collection and definitions

We collected the patient demographic characteristics, comorbidities (based on Charlson’s Comorbidity Index), reasons for ICU admission, Acute Physiology and Chronic Health Evaluation (APACHE) II and Sequential Organ Failure Assessment (SOFA) scores at ICU admission, laboratory tests (blood leukocyte count in cells/mm^3^, blood neutrophil count in cells/mm^3^, blood lymphocyte count in cells/mm^3^, blood platelet count in cells/mm^3^, blood hemoglobin in g/dL, serum D-dimer in ng/mL, serum lactate in mmol/L), microbiologic results (blood cultures, respiratory samples, and antimicrobial susceptibility), and clinical outcomes (duration of mechanical ventilation, duration of central venous catheter, ICU length of stay, ICU mortality) from the patients’ electronic medical records.

The definitions of VAP and BSI were defined according to the criteria provided by the Centers for Disease Control and Prevention’s National Healthcare Safety Network and European Centers for Disease Control and Prevention, which include clinical, radiologic, and bacteriologic criteria. Only VAP with positive microbiologic criteria was included [9, 10].

### Outcomes

The primary outcome of our study was the incidence of VAP and BSIs among patients admitted to the ICU during the COVID-19 pandemic as compared with before the pandemic. The secondary endpoint was the etiology of VAP and BSI.

### Statistical analysis

Physicians randomly selected the patients who were hospitalized in ICUs from their hospitals’ electronic data archives according to the exclusion criteria and recorded the relevant information in the prepared Microsoft Access-based data program. Patients were divided into groups based on the date of infection relative to COVID-19 as pandemic and pre-pandemic cohorts. We used Fisher’s exact test and the 2-tailed Wilcoxon signed-rank test for analysis. A *P* value of <.05 was considered statistically significant. Calculations, figures, and tables were made using the R program.

## Results

A total of 558 patients who required intensive care from six centers were included in the study. Of these patients, 422 (males (62%), whose mean age was 70 [IQR, 58–79] years) were followed up during the COVID period, and 136 (males (57%), whose mean age was 73 [IQR, 61–82] years) were followed up in the pre-COVID period. Demographic, basic clinical scales, rates of BSI and VAP, and in-hospital mortality rates in the pre-COVID and COVID periods are summarized in Table 1. The comparison of the underlying disease spectrum of the patients is also described in Table 1. Underlying disease was detected in 94% of the patients in the pre-COVID period and 92.6% of the patients in the COVID period. Hypertension was the most common underlying disease in the pre-COVID and COVID periods. It is noteworthy that during the COVID period, those with chronic lung disease were hospitalized at a lower rate and those with diabetes mellitus were hospitalized at a higher rate.

**Table 1:** Demographic, baseline, and outcome characteristics of the study population

Variables	Hospitalization periods		*P *value^2^
	Pre-COVID, *N* = 136^a^	COVID, *N* = 422	
Gender:			0.3
Male	77 (57%)	261 (62%)	
Female	59 (43%)	161 (38%)	
Age	73 (61, 82)	70 (58, 79)	0.038
APACHE II score	20 (14, 25)	17 (11, 24)	0.012
SOFA score	6.0 (4.0, 8.0)	6.0 (4.0, 8.0)	0.2
VAP	27 (20%)	86 (20%)	0.9
BSI	15 (11%)	62 (15%)	0.3
Diabetes mellitus	33 (25%)	143 (35%)	0.03
Hypertension	72 (54%)	231 (56%)	0.6
Chronic lung disease	42 (31%)	96 (23%)	0.06
Chronic kidney disease	19 (14%)	59 (14%)	0.9
Chronic heart failure	47 (35%)	134 (33%)	0.6
Solid organ tumor	21 (16%)	35 (8.5%)	0.017
Outcome			<0.001
Discharged	65 (48%)	131 (31%)	
Died	71 (52%)	291 (69%)	

^a^*N* (%); median (IQR)

^2^Pearson’s chi-squared test; Wilcoxon rank sum test

The mean APACHE II score was found as 20 (IQR, 14–25) in the pre-COVID period, which was significantly higher than in the COVID period [17 (IQR, 11–24)]. The SOFA score, on the other hand, was found to be the same in each group, with a value of 6 (IQR 4.0–8.0).

BSI and VAP rates were 20.7 (19 events in 916 patient days) and 17 (74 events in 4361 patient days) with a −3.8 difference (*P* = 0.463), and 33.7 (31 events in 919 patient days) and 34.6 (93 events in 2685 patient days) with a 0.9 difference (*P* = 0.897), respectively.

The microbiology of the infections is described in Table 2. The most frequent bacterium among patients with primary BSI in the COVID period was *Acinetobacter baumannii* (*n* = 14, 36%); this was isolated in endotracheal aspirate (ETA) cultures in both periods. *Stenotrophomonas maltophilia* was an emergent bacterium that was isolated from ETA cultures only during the COVID period.

**Table 2:** Isolated pathogens associated with specific infections at COVID and pre-COVID periods

**Infection**	**Bacterium**	**COVID period**	**Pre-COVID period**
**BSI**	*Acinetobacter baumannii*	14 (36)	2 (18)
	*Enterococcus faecium*	11 (28)	2 (18)
	*Staphylococcus hominis*	14 (36)	0 (0)
**VAP**	*Acinetobacter baumannii*	54 (62)	15 (62)
	*Klebsiella pneumoniae*	9 (10)	5 (21)
	*Pseudomonas aeruginosa*	15 (17)	4 (17)
	*Stenotrophomonas maltophilia*	9 (10)	0 (0)

## Discussion

Our retrospective cohort study found that the mean age of patients in the ICUs was lower during the COVID period. Male sex was dominant in both periods. The BSI rate increased, but the VAP rate remained stable.

Comparing the patients admitted to the ICU, we found increased mortality rates in patients admitted during the pandemic (52% vs. 69%, *P* < 0.001). Unterberg et al. also found a significant difference in the mortality rates of patients with sepsis as 52% during the pandemic and 33% before the pandemic [11]. They speculated that the patients admitted to the hospital with more severe disease were due to their delayed admission. They supported their theory with higher initial SOFA scores during the pandemic than before the pandemic. In our study, the initial mean SOFA scores were the same, but APACHE II scores were lower in patients during the COVID period. The statistically significantly increased diabetes mellitus rates may be associated with steroid treatment effects, which are widely used in pre-ICU clinics.

In their review, Fumagalli et al. reported that the VAP incidence varied from 18 to 45 episodes per 1000 ventilator days among patients with COVID-related acute respiratory distress syndrome (ARDS). Incidence rates varied widely based on the country, study population, and criteria used to identify VAP [12]. In the present study, there was no significant difference in VAP incidence density during the pandemic compared with the pre-pandemic period. Similar to ours, in a multicenter study evaluating 21 Brazilian hospitals, there was no significant difference in VAP incidence density between the two periods [13]. However, in two other recent reports, there was a significant increase in VAP rates in patients with COVID-19. In these studies, VAP rates in the control groups were 15.4 and 13 episodes per 1000 ventilator days, respectively. In our study, the high incidence rate of VAP in the control group could have affected the results [14, 15]. During the pandemic and pre-pandemic periods, the most common infections in patients who were mechanically ventilated were VAP, and in both periods, Gram-negative bacteria were the predominant microorganisms (>70% in most series) [12]. In a multicenter study conducted in Italian hub hospitals, *Enterobacterales* and *Staphylococcus aureus*caused 33% and 28% of cases of VAP, respectively, in critically ill patients with COVID-19 [16]. In a European multicenter study, Gram-negative bacilli, mainly *Pseudomonas aeruginosa* (22.3%), *Enterobacter* spp. (18.8%), and *Klebsiella*spp. (11.5%), were the most common pathogens of ventilator-associated lower respiratory tract infections in patients with COVID-19 [17]. In a Turkish study investigating the lower respiratory tract infections of patients in the ICU during the pandemic versus the pre-pandemic period, *A. baumannii* was the most common bacteria, followed by *K. pneumoniae*in both periods [18]. In our study, similar to this result, *A. baumannii* was the most common microorganism in the two periods, but *P. aeruginosa* was found to be the second most common pathogen in the COVID period and *K. pneumoniae* in the pre-COVID period. In Turkey, *A. baumannii* was an important cause of hospital-acquired infections in the ICU before the pandemic.

In our study, the BSI rates were found as 15% and 11% in the COVID and pre-COVID periods, respectively. *A. baumannii* (36%), coagulase (coA)-negative *Staphylococcus* (36%), and *Enterococcus faecium* (28%) in blood culture were the most common microorganisms during the pandemic.

Similar results were observed in a Serbian study in the ICU; *A. baumannii* (26.5%) and coA-negative *Staphylococcus* (26.5%) were the main pathogens of BSIs, followed by *Enterococcus*spp. (11.2%) [19]. Consistent with our study, Palanisamy et al. found that *A. baumannii*(32.8%) was the most common pathogen of BSIs. However, although Gram-negative pathogens (82.8%) were predominant in their study, conflictingly 64% of BSIs were Gram-positive bacteria in our study [20]. In the study of Zhu et al., 26.9% of bacteremia were in the ICU. In microbiologic analysis, mostly *Enterococcus* spp. were detected (*n*=90, 22.8%), followed by *Escherichia coli* (*n*=55, 14.0%), *Klebsiella* spp. (*n*=49, 12.4%), and CoA-negative *Staphylococcus* (*n*=48, 12.2%), respectively. *Acinetobacter*has never been revealed in blood cultures [21]. In our cohort, *Acinetobacter* spp. and *S. hominis* were found most commonly in blood cultures as associated with BSIs, followed by *E. faecium*. Interestingly, *Acinetobacter*spp. were never detected in another cohort study [3]. There were no differences in BSI- and VAP-associated etiologic patterns between patients who were polymerase chain reaction (PCR) positive and negative in the COVID period.

## Conclusions

The mortality rates of patients in the ICUs were higher during the COVID-19 period. Protective measures that prioritize healthcare workers rather than patients and exceed standard measures made no difference in terms of reducing mortality.

## Data Availability

The datasets used and/or analyzed during the current study are available from the corresponding author on reasonable request.

## Abbreviations

COVID-19: Coronavirus disease 2019
VAP: Ventilator-associated pneumonia
BSI: Bloodstream infection
ICU: Intensive care unit
IL: Interleukin
APACHE: Acute Physiology and Chronic Health Evaluation
SOFA: Sequential Organ Failure Assessment
ETA: Endotracheal aspirate
ARDS: Acute respiratory distress syndrome
PCR: Polymerase chain reaction
